# Gut health predictive indices linking gut microbiota dysbiosis with healthy state, mild gut discomfort, and inflammatory bowel disease phenotypes using gut microbiome profiling

**DOI:** 10.1128/spectrum.00271-25

**Published:** 2025-06-25

**Authors:** Joann Phan, Suneer Jain, Jurgen F. Nijkamp, Rajkumar Sasidharan, Ashish Agarwal, Julia K. Bird, Anneleen Spooren, Jonas Wittwer Schegg, Emiel Ver Loren van Themaat, Tim N. Mak

**Affiliations:** 1 Sun Genomics, Inc., San Diego, California, USA; 2 dsm-firmenich, Delft, The Netherlands; 3 Solvuu, Inc., New York, New York, USA; 4 Bird Scientific Writing, Wassenaar, The Netherlands; 5 dsm-firmenich 661754 , Kaiseraugst, Switzerland; University of Arkansas for Medical Sciences, Little Rock, Arkansas, USA; International Centre for Diarrhoeal Disease Research, Dhaka, Bangladesh

**Keywords:** gut microbiome, inflammatory bowel disease, gut inflammation, keystone score, metagenomic aerotolerant predominance index

## Abstract

Despite the complexity of the gut microbiome, several scores that use taxonomic characteristics exist that attempt to identify a healthy gut or gastrointestinal disease. Two systems in use are the metagenomic aerotolerant predominance index (MAPI) and keystone scores. The aim of this analysis was to compare different gut microbiome scores, specifically MAPI and a keystone species score, on two cross-sectional data sets and to investigate correlations of these scores with self-reported gut discomfort and gastrointestinal disease. The first data set is a commercial data set (Sun Genomics data set) with whole-genome shotgun sequencing samples from 5,372 customers. The second data set is curated from publicly available data (public data set) with 2,415 samples from participants in human studies with gut-related taxonomic profiles. MAPI scores and keystone species scores were calculated using standard methodology. The MAPI score was significantly lower in men for the public data set. There was a graded response for both the MAPI and keystone scores between healthy subjects, subjects with mild gastrointestinal discomfort, and patients with gastrointestinal disease: the MAPI score was higher, and the keystone score was lower in subjects with gastrointestinal discomfort or with inflammatory bowel disease patients. The keystone and MAPI scores have the potential to help identify factors associated with gut microbial dysbiosis and gastrointestinal discomfort or disease. Furthermore, given the functional link of the MAPI score to oxidative stress in the microbiome, the scores can help to identify conditions where oxidative stress is one of the hallmarks of dysbiosis.

**IMPORTANCE:**

Gut bacteria play a role in both mild gastrointestinal discomfort, which includes bloating and constipation, and inflammatory bowel disease. There are many different types of bacteria in the gut, and gut microbiome composition differs greatly between different people. Therefore, it is difficult to predict who has a gut microbiome associated with a healthy gut and who might develop disease or experience gut discomfort. Several scoring systems have been developed to categorize gut health states. This analysis compared two different scoring systems using data from two different sources to see how well they could identify people with gastrointestinal disease, gastrointestinal complaints, or a healthy gut. The scoring systems showed similar trends according to gut health status: groups of people with gut bacteria imbalance or gut disease had a different score than groups of people with healthy gut bacteria.

## INTRODUCTION

The complex microbial communities in the human digestive tract known as the gut microbiota are intricately involved in human health and disease (1). Considerable research has focused on the direct effects of the gut microbiome on gastrointestinal health, with emphasis on disease states and their association with microbial dysbiosis. A result of that research is the concept of gut microbiome homeostasis associated with health: disease or other gastrointestinal symptoms are thought to arise from an imbalance of the gut microbiome or an increased abundance of pathogens (2). Dysbiosis is implicated in mild gastrointestinal conditions, such as irritable bowel syndrome (IBS; [3]) and inflammatory bowel disease (IBD; [4]). There is nevertheless considerable uncertainty in the role of dysbiosis in human health: microbial imbalance of the gut has not unequivocally been shown to be a cause of disease and not a result of poor health. Moreover, there is a need to move beyond the demonstration of dysbiosis and toward the development of predictive tests or treatments for microbiome-related disease (5).

Diagnostic tests based on stool microbiome samples could potentially assist in the detection of microbial dysbiosis or to identify microbial features associated with gastrointestinal discomfort or gastrointestinal disease. Many different indices are currently under development that could use gut microbiome profiles to gauge the health and functionality of the microbiome and the potential for the development of disease (2). Indices based on alpha diversity are already associated with gastrointestinal disease status, such as the commonly used Shannon index (6), which considers the number of organisms within the ecosystem and the uniformity of population size of each species (7). There are several other indices of note that are currently under development that aim to bridge gut composition to functionality and health or disease: the metagenomic aerotolerant predominance index (MAPI) and keystone scores are two examples of these.

The metagenomic aerotolerant predominance index was originally created by linking the gut redox potential to acute malnutrition (8). It is defined as the natural logarithm of the ratio of the relative abundance of aerotolerant to strict anaerobic species (8). A MAPI score greater than 0 indicates predominance of aerobes, while a negative score is associated with a higher relative abundance of anaerobes. Various publications examining gut dysbiosis have shown overgrowth of aerotolerant or facultative aerobic species as a sign of gut microbiome dysbiosis and gastrointestinal disorders (9). For example, gut microbiome dysbiosis was observed following antibiotic treatment characterized by aerobic *Salmonella*-induced gastroenteritis. Antibiotics induced a depletion of butyrate-producing Clostridia, which is associated with negative effects on gut homeostasis (10–12). Another example is the overgrowth of commensal *Escherichia coli* in conditions of intestinal inflammation following host-driven inflammatory responses that generate reactive oxygen and nitrogen species, which conferred a growth advantage to *E. coli* (13). However, the complexity of the gut microbiome means that it is difficult to translate measures of diversity or the presence of certain microbes to a robust score.

Similarly, the keystone species approach uses a limited number of eight common strictly anaerobic gut commensals to define a healthy gut microbiome (14, 15). Under dysbiosis of the gut microbiota, certain functions exerted by the microbiome are lost, such as adequate gut barrier function, resulting in adverse effects on host health (16). Keystone species are postulated to be species that carry unique functions that are essential for the balance of the gut microbiota (15). These species exert a beneficial effect on gut balance and functionality; they are acting as “ecosystem engineers,” and when lost, they disproportionally lead to poorer gut health (15). Identifying keystone species functions across health and gastrointestinal disease could help to identify targeted interventions to selectively support their growth, functionality, and abundance in the gut microbiome ecosystem.

The ability of pathogens to cause dysbiosis might be linked at least, in part, to their ability to deal better with oxidative stress than commensal anaerobes. Indeed, 8 among the 12 WHO-listed antibiotic-resistant priority pathogens are facultative aerobes (17). The ability of pathogens to trigger metabolic pathways that allow them to handle oxidative stress might have given pathogenic species an evolutionary advantage, allowing them to outgrow commensal anaerobes in circumstances of competition for limited resources in the gut or to “hijack” the host inflammatory response, including oxidative stress, to their advantage. Conversely, the ability of commensal anaerobes to deal with oxidative stress could contribute to health and gut homeostasis (18). A recent example includes the correlation of the presence of gut-microbiome-resident antioxidant systems to health and longevity in centenarians in the Jiaoling area in China (19). Another example is the association observed between response to beta-fructan fibers in ulcerative colitis patients, where patients with an absence of gut microbiome production of riboflavin, a key redox-active and antioxidant vitamin in the gut, exhibited a proinflammatory response to beta-fructan fibers (20, 21).

These diverse examples illustrate the potential importance of functional oxidative stress response pathways in commensal anaerobes and the balance between aerotolerant and strict anaerobic species for maintaining microbiome homeostasis. While the focus has been mostly on metagenomic and metabolomic output in gut microbiome research (22), the understanding of the role of the gut environment and oxidation levels in gut homeostasis and dysbiosis remains limited. Moreover, redox balance is also linked to bioenergetic flows and cross-feeding in the microbiome (23), and therefore, any changes in redox balance or oxidative stress could trigger systemic effects on the gut microbiome ecosystem via changing the cross-feeding flows (18). It is well known from research into human homeostasis that the adaptive response to oxidative stress is one of the characteristics underlying homeostasis (24), but the understanding of the oxidative stress response in the gut microbiome and its role in gut microbiome homeostasis remains more limited (25). Elucidating the correlation of imbalance between facultative aerobe and strict anaerobic species, as described by the MAPI index on the one hand (8) and gastrointestinal discomfort and disease on the other, could be of special interest.

Therefore, the MAPI index and keystone species can be seen as two distinct markers of gut dysbiosis. Our research aimed to further map how these markers could link gut dysbiosis to gut discomfort and gastrointestinal disease. First, we investigated the use of several gut microbiome indices, derived from metagenomic analyses, to differentiate between healthy, mild gut discomfort and diseased populations, within major demographic determinants using publicly available and commercial data sets. Second, we compared the performance of the indices with each other.

The aims of further characterizing gut health indices in large-scale population cohorts, as described here, are (i) establishing characteristics that can be correlated to mild gut discomfort or gastrointestinal disease, (ii) identifying characteristics that could be modulated by interventions to restore health and gut homeostasis, and (iii) deepening our understanding of fundamental modes of action and mechanistic changes that underlie gut dysbiosis and gastrointestinal discomfort and disease.

## RESULTS

### Sun Genomics customer database analysis

In total, 5,372 profiles were used for the analysis, and demographic information is presented in Table 1. More participants were female than male, and gender was unknown for one-fifth of participants. Most participants were adults aged between 21 and 60 years, with a small proportion of children and adolescents, or seniors. Participants were from 40 countries overall; however, most participants were located in the US.

**TABLE 1:** Sun Genomics customer and public database demographics

Variable	*N*	%	*N*	%
Data set	Sun Genomics		Public data set	
Total number of participants	5,372		2,067	
Gender—male	1,979	36.8%	1,075	52%
Gender—female	2,673	49.7%	441	21.3%
Gender—not stated (%)	1,112	20.6%	551	13.5%
Age 0–10 yr	602	11.2%	203	9.8%
Age 11–20 yr	331	6.2%	425	20.6%
Age 21–30 yr	640	11.9%	287	13.9%
Age 31–40 yr	1,213	22.6%	558	27%
Age 41–50 yr	1,045	19.5%	10	0.5%
Age 51–60 yr	846	15.7%	51	2.5%
Age 61–70 yr	476	8.9%	63	3%
Age 71–80 yr	189	3.5%	16	0.8%
Age 81+ yr	30	0.6%	–	–
Country—US	4,909	91.4%	–	–
Country—UK	162	3.0%	–	–
Country—other	301	5.6%	–	–

**Fig 1:**
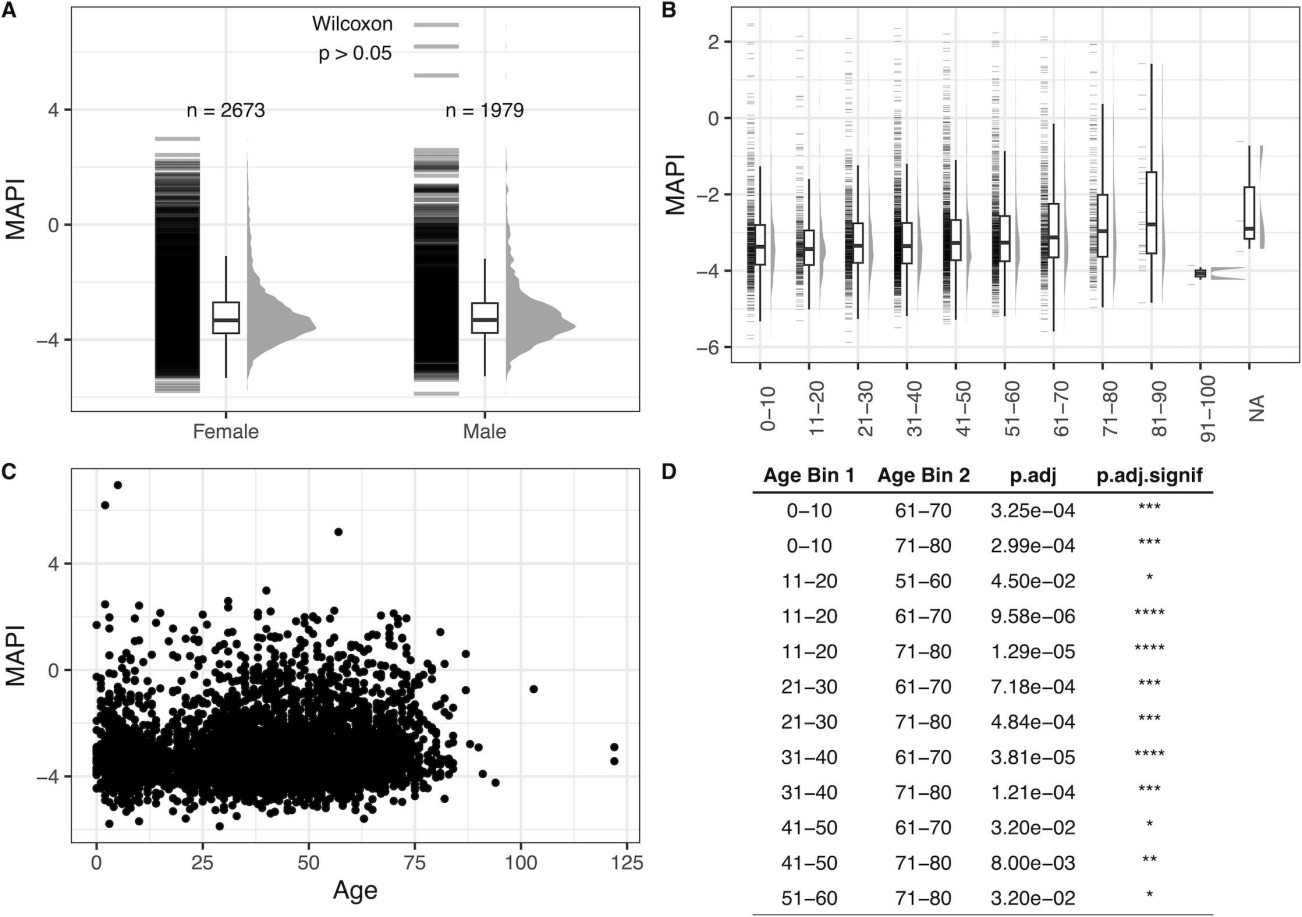
Age, gender, and MAPI distribution in the Sun Genomics data set. (**A**) Gender distribution of MAPI score in timepoint one profiles, (**B**) boxplots of age bins, (**C**) distribution of MAPI score by age, and (**D**) MAPI distribution across age bins. Wilcoxon tests were performed to test significance between MAPI scores across age bins. *P*-values were corrected for multiple comparisons with False Discovery Rate (FDR) for the results in D.

MAPI score was analyzed according to gender and age categories in the Sun Genomics data set (Fig. 1). The average MAPI score was not significantly different in male compared to female participants (Wilcoxon signed-rank test, *P* > 0.05; Fig. 1A). MAPI score increased with age (Fig. 1B and C), and there were significant differences found between different age categories (Fig. 1D). Namely, the MAPI scores of age category 1–10 were significantly lower than 61–80 years, 11–20 years were significantly lower than 51–80 years, and age category 21–60 years was significantly lower than the age category 61–80 years. The small sample size for the 91–100-year category prevents generalizations from being made about this age group.

**Fig 2:**
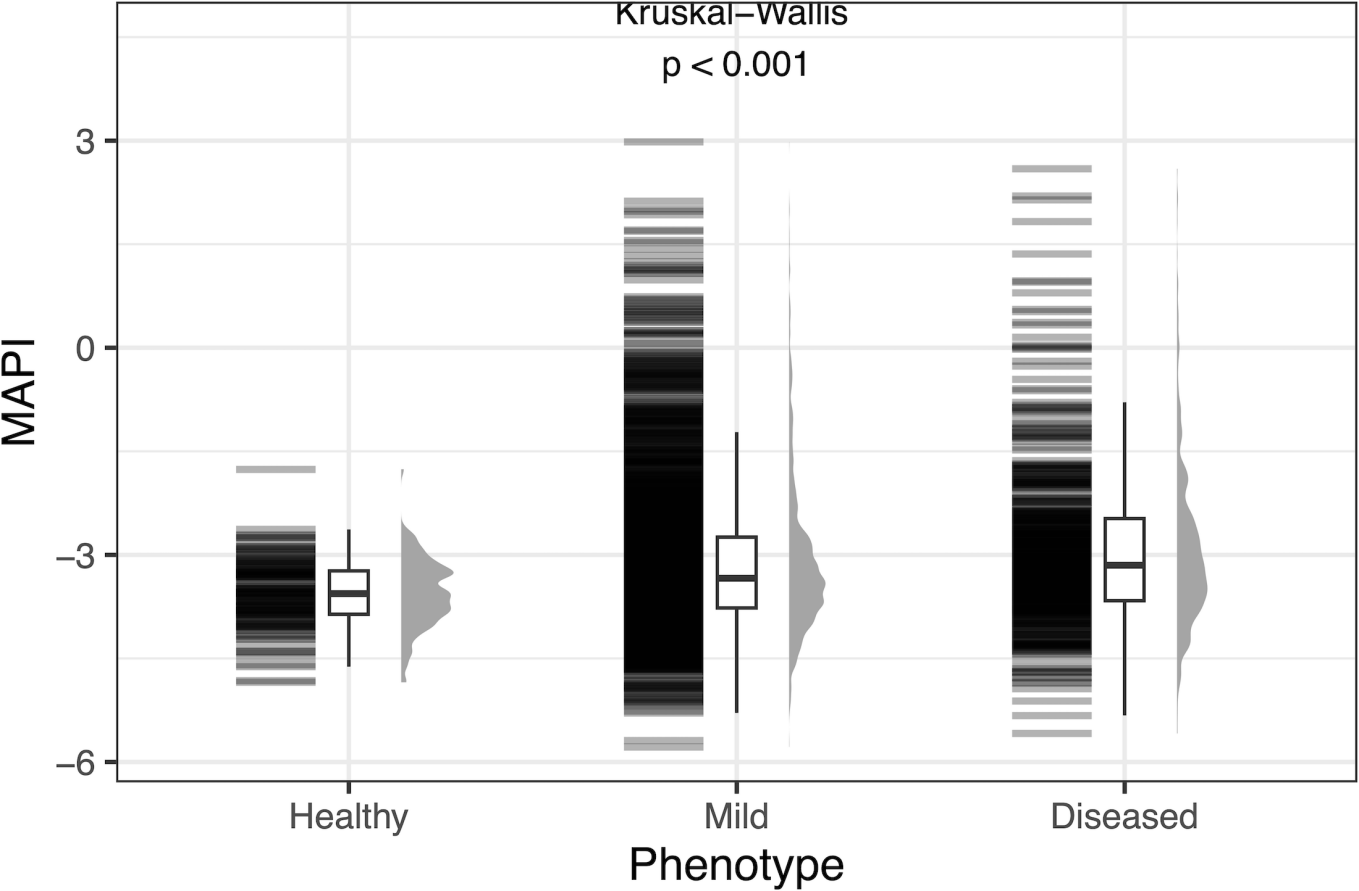
MAPI score across healthy, mild, and diseased phenotypes in the Sun Genomics data set. The Kruskal-Wallis test was used to calculate the *P*-value.

The MAPI score was compared between healthy, mild, and diseased phenotypes in Fig. 2. MAPI score was higher in the mild and disease phenotypes compared to the healthy phenotype, indicating that participants with the greatest disease burden had a slightly higher relative abundance of aerotolerant microbes. The Kruskal-Wallis test indicates that at least one phenotype stochastically dominates the other(s). When assessing the alpha and beta diversities of the profiles across phenotypic bins, there was no significant difference in alpha diversity and low separation between bins on a Principal Component Analysis (results not shown).

**Fig 3:**
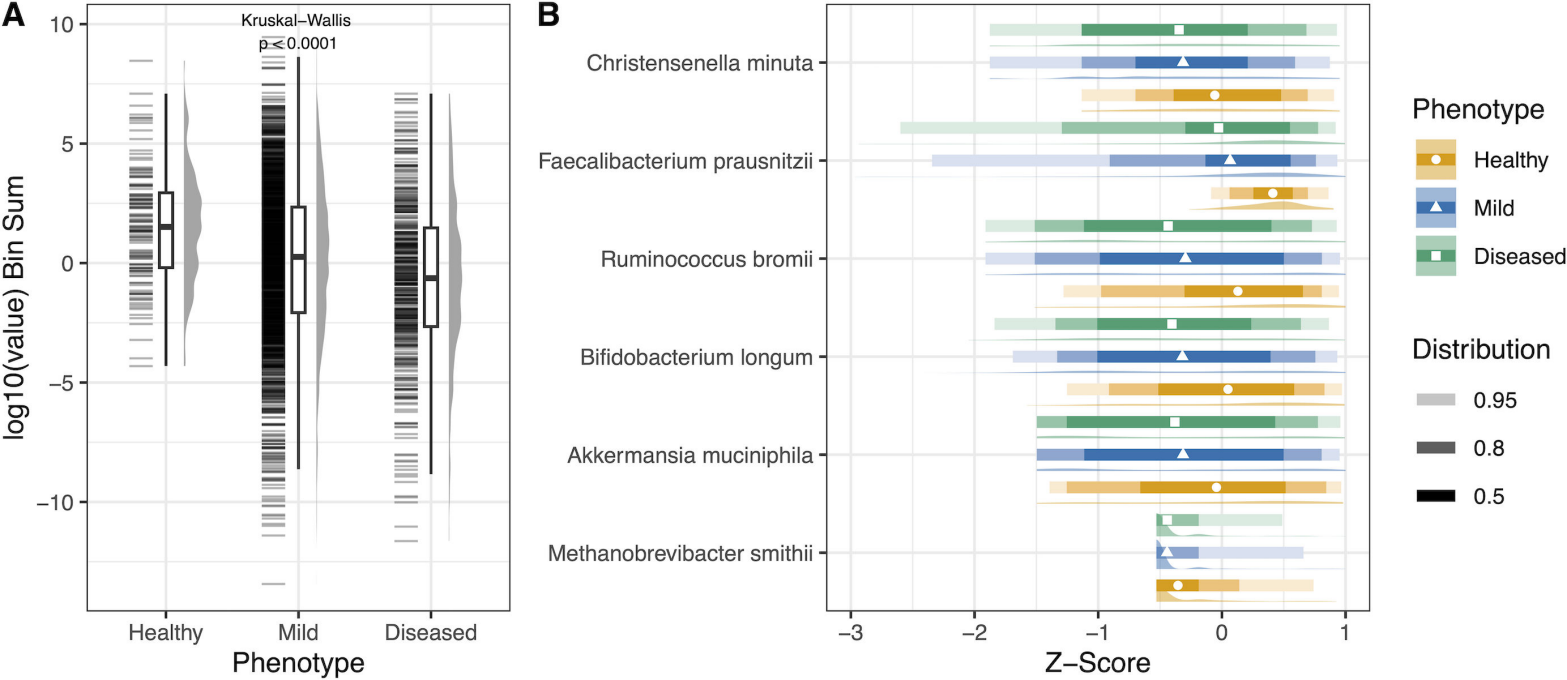
Keystone species score across phenotypic bins in the Sun Genomics data set. (**A**) Total mean keystone score by phenotype and (**B**) individual keystone species relative proportions by phenotype. Relative proportions were log10 transformed, and *Z*-scores were calculated for each of the six keystone species. The Kruskal-Wallis test was used to calculate the *P*-value.

Disease phenotypes were compared using the keystone species score and individual keystone species abundance (Fig. 3). Log_10_ bin sum of the keystone species score is decreased in the diseased phenotype compared to higher scores in the healthy phenotype, indicating that there is a higher proportion of keystone species in the healthy population and a lower proportion of keystone species in the diseased population (Fig. 3A). The relative proportion of individual keystone species was the highest in the healthy phenotype, except for *Methanobrevibacter smithii* (Fig. 3B).

### Public data set analyses

**Fig 4:**
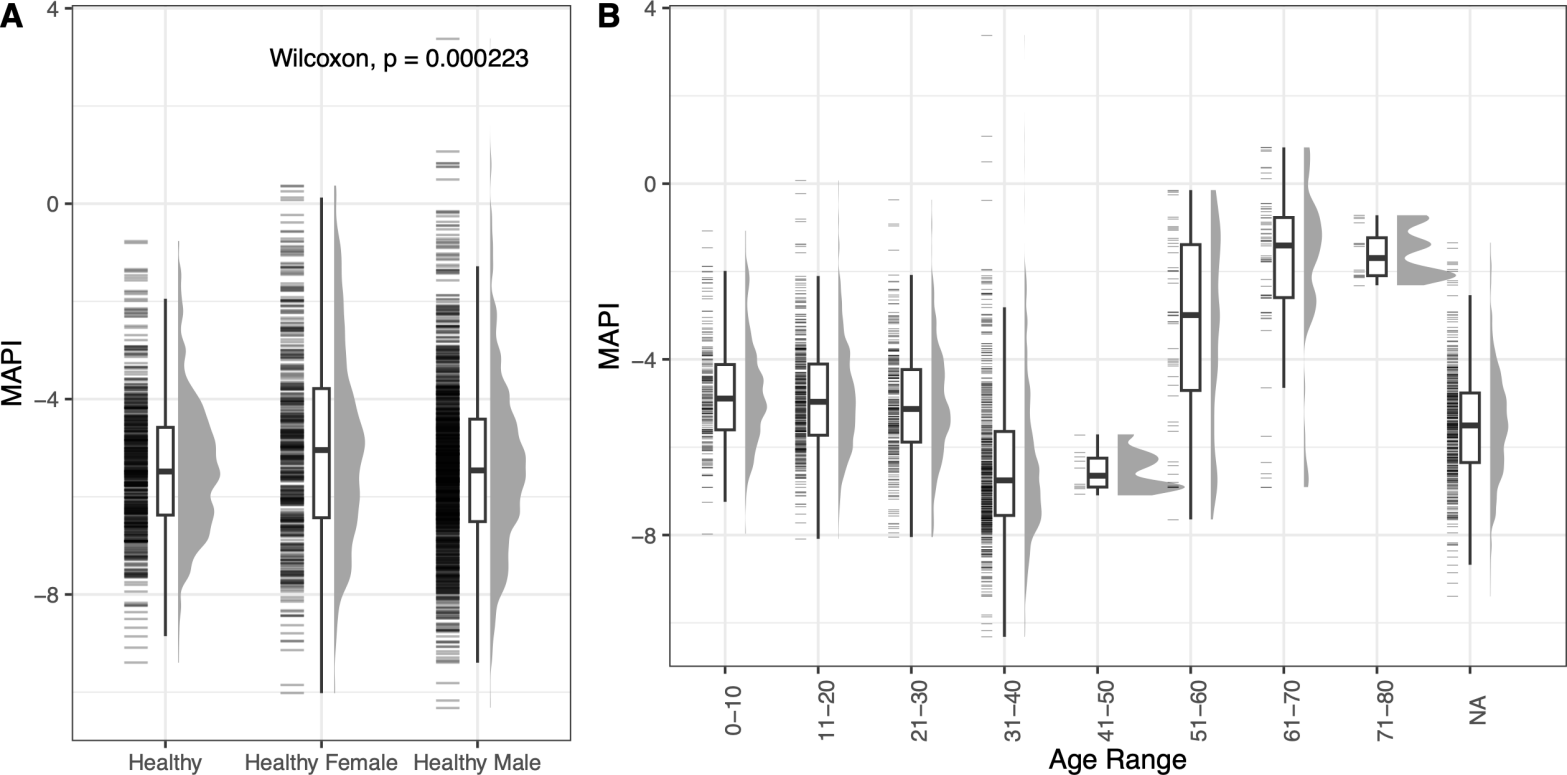
MAPI score by gender and age category in the public data set. Distribution of MAPI score by (**A**) overall healthy, healthy males, and healthy females and (**B**) age categories. The Wilcoxon test was used to calculate the *P*-value.

A total of 2,415 MAPI scores from healthy adults were collated from 8 independent studies in order to obtain an overall healthy MAPI score distribution (Table 2). From these, 1,075 MAPI scores were from male and 441 were from female participants (Table 1). MAPI score distribution in the public data sets is shown in Fig. 4. On average, the MAPI score of healthy individuals was −5.2 with an SD of 1.8. MAPI scores in healthy males were slightly but significantly lower than in females (*P* < 0.0001).

**TABLE 2:** Public data set description

Study ID	Study description	*N* ^*a*^	Database	Cohort	Reference
MGYS00005259	Prebiotic fiber supplementation in 174 healthy young adults (US)	174	MGnify	Healthy	(26)
MGYS00005601	94 Healthy controls from 10 Parkinson’s disease trials	92	MGnify	Healthy	(27)
MGYS00001175	26 Healthy young adults given an antibiotic or controls (Canada)	348	MGnify	Healthy	(28)
MGYS00005628	358 Healthy adult controls in a study with colorectal cancer cases (France, Germany, Denmark, and Spain)	288	MGnify	Healthy	(29)
MGYS00005184	Large cohort of 1,651 healthy adults, some of whom are related (Canada)	959	MGnify	Healthy	(30)
Gevers2014_IBD	Healthy controls in four pooled IBD studies	113	MicrobiomeHD	Healthy + IBD	(31)
Morgan2012_IBD			MicrobiomeHD	Healthy + IBD	(32)
Papa2012_IBD			MicrobiomeHD	Healthy + IBD	(33)
Willing2009_IBD			MicrobiomeHD	Healthy + IBD	(34)

^*a*^ Number of samples.

### Comparison of MAPI score in the Sun Genomics and public data set

**Fig 5:**
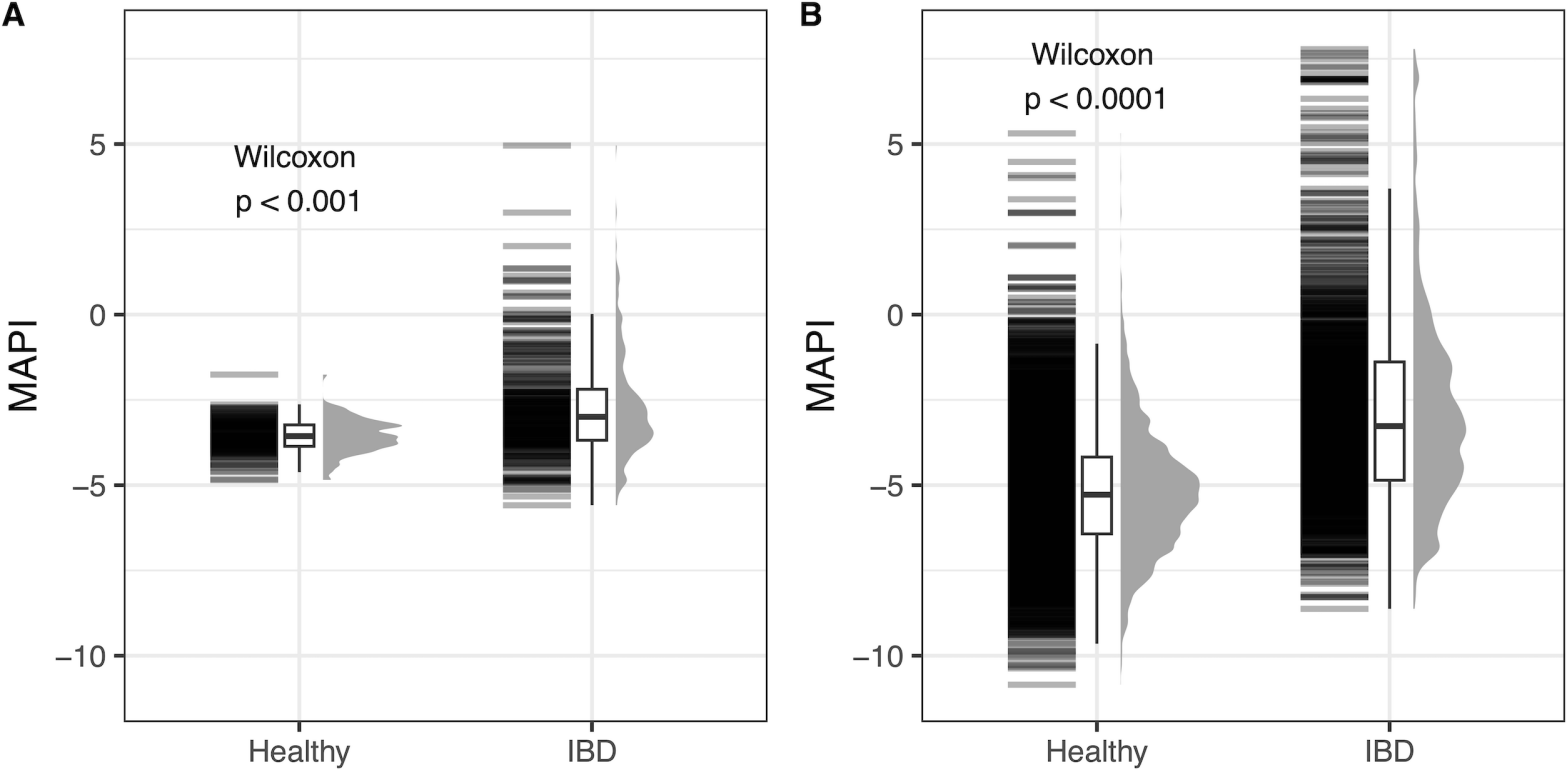
Comparison of MAPI scores between healthy and IBD subjects in the (**A**) Sun Genomics and (**B**) public data sets. MAPI scores for healthy subjects and subjects with IBD in the (**A**) Sun Genomics and (**B**) public data sets. The nonparametric Wilcoxon test was used to calculate the *P*-value.

The MAPI score in healthy subjects and patients with IBD was compared between the Sun Genomics and public data sets (Fig. 5). Both data sets showed the same significant relationship, with a higher MAPI in the IBD patients compared to healthy controls. However, the average MAPI score was different between the data sets, with a broader range found in the public data set.

## DISCUSSION

The current analysis investigated two different indices of gut microbiome health in two data sets. Changes in the scores according to demographic factors and phenotypic differences in terms of chronic gastrointestinal discomfort or disease status were also explored. Significant differences according to gender were only found in the public data set, with a lower mean MAPI score found in men, although the results here may have been skewed due to unbalanced sample inclusion. The Sun Genomics data set found a significant relationship between MAPI score and age; older participants had a higher MAPI score compared to younger age groups. Differences were found in both the MAPI and keystone scores according to disease-related phenotype, with a graded increase through healthy participants, those with mild gastrointestinal conditions, and patients with gastrointestinal disease. A difference was found between healthy subjects and those with IBD when the MAPI score was compared, which was true across data sets. Thus, we found that higher MAPI scores, representing a higher presence of aerotolerant species and possibly higher levels of oxidative stress, were indicative of gastrointestinal mild discomfort and disease, with disease states being characterized by the highest MAPI scores.

The Sun Genomics data set comprised a large number of participants obtained from the customer database; thus, the microbiome scores obtained from this data set give a good indication of the range of values that can be found under the free-living population in the US, albeit those who have the interest in and ability to purchase a microbiome analysis kit. US-based research has found socio-economic status (SES) to be a strong predictor of the gut microbiome (35), which means that the sample is not representative of the US population or their microbiota, as participants were more likely to have a higher SES. Likewise, the public data set is drawn from a diverse range of clinical trials and cohort studies conducted in various countries, including Germany, Spain, Denmark, France, US, and Canada. The results from this analysis reflect normal scores that can be obtained in surveys and gut microbiome profiles from whole-genome sequencing or amplicon sequencing.

There was no significant difference found in the MAPI score by gender in the Sun Genomics data set, while there was a significant difference found in the public data set. This may reflect bias in the sample. To illustrate, the study described by Turpin and co-authors contained a substantial number of samples from family members (30). The sampling of subjects that share environmental factors may have impacted the correlation between gender and MAPI score (36).

The relationship between MAPI score and age was shown in both data sets with an overall increase in the MAPI score in older subjects (greater than 61 years old). Additionally, the same trend was found by analyzing data from a survey of microbiome samples obtained from 367 Japanese subjects aged 0–104 years, with the highest MAPI score found in the highest age groups (37). An analysis of the genera in samples from the same cohort found a continuous progression in 36 species across the spectrum of aging, with changes in abundance at the highest and lowest age groups (38). Interestingly, this study can be linked to another recent observation of a higher presence of gut-microbiome-resident antioxidant systems in centenarians with a healthy lifespan in the Jiaoling area in China (19). Another possibility is that an increase in medication use for co-morbidities, which is common for chronic conditions in the elderly, induces changes in the microbiome that affect the MAPI score (39). For example, proton pump inhibitors used to treat gastric reflux induce dysbiosis and gastrointestinal conditions, such as small intestine bacterial overgrowth and inflammatory bowel diseases (39). These combined observations provide a basis to further investigate the role of oxidative stress and aerotolerant/strict anaerobe balance in healthy aging. In addition, chronological age may be differentiated from biological age in regard to the microbiome; indeed, microbial imbalance has been identified as a hallmark of aging (40). Metatranscriptomic data have been used to find associations between biological age and the microbiome (41). Future analyses should consider how biological age affects or is affected by the microbiome.

Disease status affected both the MAPI and keystone scores: both showed a gradual change between healthy, mild, and diseased phenotypes, although there was considerable variation, and the differences were small and did not allow individuals to be classified. Figure 2 and 3A show that both scores ranked the healthy cohort lowest, with the mild condition intermediate, and the highest score was found in the diseased cohort. Visually, variation appeared to increase in diseased participants, although this was not tested with an appropriate statistical test. In the original MAPI analysis, the healthy cohorts of French adults and Senegali children had a lower MAPI score than the patients with dysfunctional dietary status (8). The higher MAPI score could be indicative of oxidative stress and thus a cause or result of the disease condition (8). This investigation further expands the understanding of the correlation of the MAPI index and oxidative stress (9) to gastrointestinal discomfort and disorders.

Despite similar trends seen between the analyses performed on the Sun Genomics and public data sets, differences are seen in the absolute scores found, and the lack of effect by gender seen in the Sun Genomics data set. Methodological differences in the analyses presented here could be responsible for differences in absolute scores. The Sun Genomics data set used whole-genome shotgun sequencing, while the 16S rRNA gene-based and shotgun methods were used for the public data set analysis. These two methods have intrinsic differences in terms of the type and quantity of information collected on the microbiome (42, 43). To our knowledge, there has been no comparison of these two technologies on MAPI score calculation; therefore, it is difficult to specify expected differences. However, identification of bacteria to species level using whole-genome shotgun sequencing will not change the MAPI score calculation, which uses genus information.

While providing a simple way to compare microbiomes, the keystone approach has several drawbacks (44). Using a taxonomic approach does not take into account the effect of microbes that fulfill a redundant functional role in the gut environment yet are not included in the score calculation, which is based on taxonomic classification. Also, it is questionable whether the small number of species selected can account for the complexity found in gut ecosystems, especially when considering inter-individual variation (45). Some species are not included in the keystone approach despite being considered crucial for essential functions in microbiome metabolic competence, such as *Blautia* spp., which is a major electron sink (23). Future work on the keystone score could include this species, given its role within the gut microbial ecosystem. Nevertheless, the observations here linking keystone score with disease state provide a basis for further research in this area.

There are several limitations in the data sets that limit the applicability of these results to others. While we wanted to investigate gut microbiome scores within healthy populations, both data sets are drawn from populations with specific limitations. The Sun Genomics data set is drawn from customers that have purchased a service to analyze their microbiome; thus, these participants are likely to be interested in their health and have the means to incur out-of-pocket health-related costs. The demographic and health-related data presented in the Sun Genomics data set also rely completely on self-reported information by participants. A limitation of the self-reported information is the possibility of individual subjectiveness in reporting gastrointestinal health status. Approximately 20% of the participants did not report their gender, indicating that a considerable portion of the demographic data was censored through nonreport for this variable. Despite the Sun Genomics sample being predominantly from the US, the results are unlikely to be representative of the US population because healthy participants who are unable to or not interested in obtaining a microbiome sample were not included. The public data set also suffers from selection bias, including healthy subjects in more highly defined clinical trials who are global but not representative of the entire healthy population.

While this analysis provides some evidence that both the MAPI and keystone scores can partially differentiate between healthy subjects and patients with gastrointestinal symptoms and conditions, the wide variance and overlap in confidence intervals do not allow diagnosis of disease based on the current scores. Both scores rely on taxonomic classification at the genus or species level. Integrated approaches using multi-omics data and separation to entero-signatures during dysbiosis (46) may provide a more robust means to identify microbial dysbiosis that leads to disease and a functional understanding of disease-associated changes in the genome or metabolome (47). The observations on MAPI score and its link to age and gastrointestinal complaints are in line, however, with other studies that investigated the role of oxidative stress and overgrowth of aerotolerant species to microbiome dysbiosis (18).

Our analyses provide a comparison of two gut microbiome scores and how they vary according to demographics and gastrointestinal disease phenotypes. Both gut microbiome scores showed a differential effect of disease phenotype on the score, which provides a method to distill the complexity of the microbiome into a single score that is relevant to health, although this currently lacks the precision required for clinical practice. Given the general correlation between the MAPI score with redox level and oxidative stress in the intestinal environment, and the correlation of MAPI to gastrointestinal discomfort and disease, the current results suggest that the overgrowth of facultative aerobic/aerotolerant bacteria could be an underlying sign of gut microbiome dysbiosis and is weakly correlated to reported gastrointestinal complaints. Further work is needed to develop diagnostic tools for clinicians to use in diagnosing or treating gastrointestinal disease; however, it is possible that a gut microbiome-based score could be used in the future.

## MATERIALS AND METHODS

### Sun Genomics data set

#### Participants and sample collection

The basis of the Sun Genomics data set was the customer database containing cross-sectional information about age, gender, geographic location, a fecal microbiome profile, and a completed health and diet survey.

Fecal samples were taken using at-home collection components for stool with a dry and liquid-filled collection tube and a collection swab that subjects had purchased as part of the Floré Gut Health Test, as described previously (48). Briefly, participants were instructed to use the swab to collect a pea-sized sample of stool immediately after a bowel movement. Participants were asked to ensure that the stool sample was not contaminated with urine. After collection, participants shipped their samples at ambient temperature using the 2-day standard postal service delivery. Upon arrival at Floré laboratories, samples were stored at 4°C and processed within 3 days. When registering their kit, participants provided informed consent for use of data in accordance with IRB no. SG-04142018-001 and were given the opportunity to fill out a health and diet survey.

#### Microbiome analysis

Total DNA was extracted and purified using a proprietary method (patents 10428370 and 10837046). To prepare for whole-genome shotgun metagenomics, DNA libraries were prepared with NEBNext reagents and MagJet magnetic beads. Briefly, DNA was sheared, ends were repaired, adapters were ligated, and library concentrations were quantified by qPCR. After library normalization, libraries were pooled and loaded onto an Illumina NextSeq 550 using 150 × 150 bp paired-end reads. After sequencing, reads were quality filtered and processed to remove human reads. Taxonomy of the quality-filtered reads was classified using the Gutbuster platform with a hand-curated database of approximately 23,000 microbial species.

#### Selection of profiles and statistical analysis

Microbiome profiles with associated health and diet surveys from the Sun Genomics customer database were sorted into healthy, mild, and diseased populations according to self-report by participants in the Sun Genomics health and diet survey. Healthy subjects reported no health or gut issues. Mild conditions included bloating, constipation, gassiness, and IBS. Diseased conditions included Celiac disease, Crohn’s disease, gastroesophageal reflux disease, inflammatory bowel disease, and ulcerative colitis.

To test for statistical significance between cohorts, Wilcoxon rank-sum tests were performed with corrections for multiple hypotheses where applicable when multiple comparisons are tested. Analyses were performed in R version 4.2.3.

### Public data set

Two sources of public data sets were used for the public data set analysis: the European Bioinformatics Institute MGnify portal (49) and the MicrobiomeHD data set collection, to form a cross-sectional survey data set (50). The data sets are described in more detail in Table 2.

The MGnify portal was searched using one or more of the following keywords—“diet,” “GI indication,” and “supplements.” The resulting search results were further analyzed for relevance and shortlisted for data processing. Metadata were reviewed for relevance, namely that some of the samples in the study should be gut related and that metadata should contain information on participants who were healthy or had a gastrointestinal condition. Only gut-related samples in each study were downloaded. As taxonomic assignments are provided for both small subunit and large subunit, it was not clear which should be used, and therefore, the summary with the highest Operational Taxonomic Unit (OTU) count of the two was used. Analyses were performed on this data set using Python. To fit the format requirements of the *predict.py* tool, the following modifications were made: (i) removing “s” at the beginning of each OTU, (ii) removing all “Root;” occurrences, (iii) renaming “SampleID” to “OTU ID,” and (iv) replacing “;” with “|” everywhere in the file. After running *predict.py* to derive MAPI and redox scores, the number of files in the output was verified with the number of samples in the taxonomic summary file.

The article by Duvallet et al. provided the microbiomeHD database (50), consisting of 29 case control studies containing 16S rRNA gene-based microbiota data sets. Studies can be downloaded individually as *.tar.gz files with top-level identifiers per data set. We used the metadata file and the 100% OTU tables with Latin taxonomic names assigned using the naive Bayesian classifier "RDP classifier" (*c* = 0.5). After minor changes were made, *predict.py* was run on the OTU tables for each data set.

#### MAPI score calculation

For each microbiome sample, the MAPI score (8) was calculated as the natural logarithm of the ratio of the sum of the abundance of aerobic genera divided by the sum of the abundance of strict anaerobic genera. For Bifidobacteria, strict anaerobic taxa are defined at the species level. The list with strict anaerobic taxa was defined using the “List of Prokaryotes According to Their Aerotolerant or Obligate Anaerobic Metabolism” (8, 51).

#### Keystone score calculation

The keystone score was calculated based on keystone species presented by Tudela et al. (15). The six keystone species used in this analysis were *Akkermansia muciniphila*, *Bifidobacterium longum*, *Christensenella minuta*, *Faecalibacterium prausnitzii*, *Methanobrevibacter smithii*, and *Ruminococcus bromii*. *Z*-scores were calculated from log-transformed relative abundances across each species. The keystone score is calculated by summing the *Z*-scores of six keystone species for each sample.

## Supplementary Material

Reviewer comments

## Data Availability

A full implementation of the MAPI score calculation is available via Bitbucket, https://bitbucket.org/dfsbioitteam/mapi_deploy/. The public data set can be accessed using the information provided in Table 2. Data from the Sun Genomics data set cannot be made open access due to subject confidentiality; however, de-identified data may be made available after reasonable request to the authors.

## SUPPLEMENTAL MATERIAL

OPEN PEER REVIEW reviewer-comments.pdf An accounting of the reviewer comments and feedback.

ASM does not own the copyrights to Supplemental Material that may be linked to, or accessed through, an article. The authors have granted ASM a non-exclusive, world-wide license to publish the Supplemental Material files. Please contact the corresponding author directly for reuse.

## References

[1] Bull MJ, Plummer NT. 2014. Part 1: The human gut microbiome in health and disease. Integr Med (Encinitas) 13:17–22. 26770121 PMC4566439

[2] Lozupone CA, Stombaugh JI, Gordon JI, Jansson JK, Knight R. 2012. Diversity, stability and resilience of the human gut microbiota. Nature 489:220–230. doi:10.1038/nature11550 22972295 PMC3577372

[3] Enck P, Aziz Q, Barbara G, Farmer AD, Fukudo S, Mayer EA, Niesler B, Quigley EMM, Rajilić-Stojanović M, Schemann M, Schwille-Kiuntke J, Simren M, Zipfel S, Spiller RC. 2016. Irritable bowel syndrome. Nat Rev Dis Primers 2:16014. doi:10.1038/nrdp.2016.14 27159638 PMC5001845

[4] Santana PT, Rosas SLB, Ribeiro BE, Marinho Y, de Souza HSP. 2022. Dysbiosis in inflammatory bowel disease: pathogenic role and potential therapeutic targets. Int J Mol Sci 23:3464. doi:10.3390/ijms23073464 35408838 PMC8998182

[5] Olesen SW, Alm EJ. 2016. Dysbiosis is not an answer. Nat Microbiol 1:16228. doi:10.1038/nmicrobiol.2016.228 27886190

[6] Gong D, Gong X, Wang L, Yu X, Dong Q. 2016. Involvement of reduced microbial diversity in inflammatory bowel disease. Gastroenterol Res Pract 2016:6951091. doi:10.1155/2016/6951091 28074093 PMC5198157

[7] Kim BR, Shin J, Guevarra R, Lee JH, Kim DW, Seol KH, Lee JH, Kim HB, Isaacson R. 2017. Deciphering diversity indices for a better understanding of microbial communities. J Microbiol Biotechnol 27:2089–2093. doi:10.4014/jmb.1709.09027 29032640

[8] Million M, Raoult D. 2018. Linking gut redox to human microbiome. Hum Microbiome J 10:27–32. doi:10.1016/j.humic.2018.07.002

[9] de Vos WM, Tilg H, Van Hul M, Cani PD. 2022. Gut microbiome and health: mechanistic insights. Gut 71:1020–1032. doi:10.1136/gutjnl-2021-326789 35105664 PMC8995832

[10] Rivera-Chávez F, Zhang LF, Faber F, Lopez CA, Byndloss MX, Olsan EE, Xu G, Velazquez EM, Lebrilla CB, Winter SE, Bäumler AJ. 2016. Depletion of butyrate-producing Clostridia from the gut microbiota drives an aerobic luminal expansion of Salmonella. Cell Host Microbe 19:443–454. doi:10.1016/j.chom.2016.03.004 27078066 PMC4832419

[11] Winter SE, Thiennimitr P, Winter MG, Butler BP, Huseby DL, Crawford RW, Russell JM, Bevins CL, Adams LG, Tsolis RM, Roth JR, Bäumler AJ. 2010. Gut inflammation provides a respiratory electron acceptor for Salmonella. Nature 467:426–429. doi:10.1038/nature09415 20864996 PMC2946174

[12] Guo P, Zhang K, Ma X, He P. 2020. Clostridium species as probiotics: potentials and challenges. J Anim Sci Biotechnol 11:24. doi:10.1186/s40104-019-0402-1 32099648 PMC7031906

[13] Winter SE, Winter MG, Xavier MN, Thiennimitr P, Poon V, Keestra AM, Laughlin RC, Gomez G, Wu J, Lawhon SD, Popova IE, Parikh SJ, Adams LG, Tsolis RM, Stewart VJ, Bäumler AJ. 2013. Host-derived nitrate boosts growth of E. coli in the inflamed gut. Science 339:708–711. doi:10.1126/science.1232467 23393266 PMC4004111

[14] Banerjee S, Schlaeppi K, van der Heijden MGA. 2018. Keystone taxa as drivers of microbiome structure and functioning. Nat Rev Microbiol 16:567–576. doi:10.1038/s41579-018-0024-1 29789680

[15] Tudela H, Claus SP, Saleh M. 2021. Next generation microbiome research: identification of keystone species in the metabolic regulation of host-gut microbiota interplay. Front Cell Dev Biol 9:719072. doi:10.3389/fcell.2021.719072 34540837 PMC8440917

[16] Hrncir T. 2022. Gut microbiota dysbiosis: triggers, consequences, diagnostic and therapeutic options. Microorganisms 10:578. doi:10.3390/microorganisms10030578 35336153 PMC8954387

[17] André AC, Debande L, Marteyn BS. 2021. The selective advantage of facultative anaerobes relies on their unique ability to cope with changing oxygen levels during infection. Cell Microbiol 23:e13338. doi:10.1111/cmi.13338 33813807

[18] Kunst C, Schmid S, Michalski M, Tümen D, Buttenschön J, Müller M, Gülow K. 2023. The influence of gut microbiota on oxidative stress and the immune system. Biomedicines 11:1388. doi:10.3390/biomedicines11051388 37239059 PMC10216031

[19] Wu L, Xie X, Li Y, Liang T, Zhong H, Yang L, Xi Y, Zhang J, Ding Y, Wu Q. 2022. Gut microbiota as an antioxidant system in centenarians associated with high antioxidant activities of gut-resident Lactobacillus. NPJ Biofilms Microbiomes 8:102. doi:10.1038/s41522-022-00366-0 36564415 PMC9789086

[20] Bourgonje AR. 2023. Microbial riboflavin biosynthesis associates with response to dietary fiber consumption: time to personalize adjunct therapy in patients with inflammatory bowel disease? Gastroenterology 165:515–516. doi:10.1053/j.gastro.2022.10.016 36279921

[21] Armstrong HK, Bording-Jorgensen M, Santer DM, Zhang Z, Valcheva R, Rieger AM, Sung-Ho Kim J, Dijk SI, Mahmood R, Ogungbola O, et al.. 2023. Unfermented β-fructan fibers fuel inflammation in select inflammatory bowel disease patients. Gastroenterology 164:228–240. doi:10.1053/j.gastro.2022.09.034 36183751

[22] Puig-Castellví F, Pacheco-Tapia R, Deslande M, Jia M, Andrikopoulos P, Chechi K, Bonnefond A, Froguel P, Dumas M-E. 2023. Advances in the integration of metabolomics and metagenomics for human gut microbiome and their clinical applications. Trends Anal Chem 167:117248. doi:10.1016/j.trac.2023.117248

[23] Daisley BA, Koenig D, Engelbrecht K, Doney L, Hards K, Al KF, Reid G, Burton JP. 2021. Emerging connections between gut microbiome bioenergetics and chronic metabolic diseases. Cell Rep 37:110087. doi:10.1016/j.celrep.2021.110087 34879270

[24] Pickering AM, Vojtovich L, Tower J, A. Davies KJ. 2013. Oxidative stress adaptation with acute, chronic, and repeated stress. Free Radic Biol Med 55:109–118. doi:10.1016/j.freeradbiomed.2012.11.001 23142766 PMC3687790

[25] Sun Y, Wang X, Li L, Zhong C, Zhang Y, Yang X, Li M, Yang C. 2024. The role of gut microbiota in intestinal disease: from an oxidative stress perspective. Front Microbiol 15:1328324. doi:10.3389/fmicb.2024.1328324 38419631 PMC10899708

[26] Baxter NT, Schmidt AW, Venkataraman A, Kim KS, Waldron C, Schmidt TM. 2019. Dynamics of human gut microbiota and short-chain fatty acids in response to dietary interventions with three fermentable fibers. MBio 10:e02566-18. doi:10.1128/mBio.02566-18 30696735 PMC6355990

[27] Romano S, Savva GM, Bedarf JR, Charles IG, Hildebrand F, Narbad A. 2021. Meta-analysis of the Parkinson’s disease gut microbiome suggests alterations linked to intestinal inflammation. npj Parkinsons Dis 7:27. doi:10.1038/s41531-021-00156-z 33692356 PMC7946946

[28] Raymond F, Ouameur AA, Déraspe M, Iqbal N, Gingras H, Dridi B, Leprohon P, Plante P-L, Giroux R, Bérubé È, Frenette J, Boudreau DK, Simard J-L, Chabot I, Domingo M-C, Trottier S, Boissinot M, Huletsky A, Roy PH, Ouellette M, Bergeron MG, Corbeil J. 2016. The initial state of the human gut microbiome determines its reshaping by antibiotics. ISME J 10:707–720. doi:10.1038/ismej.2015.148 26359913 PMC4817689

[29] Zeller G, Tap J, Voigt AY, Sunagawa S, Kultima JR, Costea PI, Amiot A, Böhm J, Brunetti F, Habermann N, et al.. 2014. Potential of fecal microbiota for early-stage detection of colorectal cancer. Mol Syst Biol 10:766. doi:10.15252/msb.20145645 25432777 PMC4299606

[30] Turpin W, Espin-Garcia O, Xu W, Silverberg MS, Kevans D, Smith MI, Guttman DS, Griffiths A, Panaccione R, Otley A, Xu L, Shestopaloff K, Moreno-Hagelsieb G, GEM Project Research Consortium, Paterson AD, Croitoru K. 2016. Association of host genome with intestinal microbial composition in a large healthy cohort. Nat Genet 48:1413–1417. doi:10.1038/ng.3693 27694960

[31] Gevers D, Kugathasan S, Denson LA, Vázquez-Baeza Y, Van Treuren W, Ren B, Schwager E, Knights D, Song SJ, Yassour M, et al.. 2014. The treatment-naive microbiome in new-onset Crohn’s disease. Cell Host Microbe 15:382–392. doi:10.1016/j.chom.2014.02.005 24629344 PMC4059512

[32] Morgan XC, Tickle TL, Sokol H, Gevers D, Devaney KL, Ward DV, Reyes JA, Shah SA, LeLeiko N, Snapper SB, Bousvaros A, Korzenik J, Sands BE, Xavier RJ, Huttenhower C. 2012. Dysfunction of the intestinal microbiome in inflammatory bowel disease and treatment. Genome Biol 13:R79. doi:10.1186/gb-2012-13-9-r79 23013615 PMC3506950

[33] Papa E, Docktor M, Smillie C, Weber S, Preheim SP, Gevers D, Giannoukos G, Ciulla D, Tabbaa D, Ingram J, Schauer DB, Ward DV, Korzenik JR, Xavier RJ, Bousvaros A, Alm EJ. 2012. Non-invasive mapping of the gastrointestinal microbiota identifies children with inflammatory bowel disease. PLoS ONE 7:e39242. doi:10.1371/journal.pone.0039242 22768065 PMC3387146

[34] Willing BP, Dicksved J, Halfvarson J, Andersson AF, Lucio M, Zheng Z, Järnerot G, Tysk C, Jansson JK, Engstrand L. 2010. A pyrosequencing study in twins shows that gastrointestinal microbial profiles vary with inflammatory bowel disease phenotypes. Gastroenterology 139:1844–1854. doi:10.1053/j.gastro.2010.08.049 20816835

[35] Kwak S, Usyk M, Beggs D, Choi H, Ahdoot D, Wu F, Maceda L, Li H, Im EO, Han HR, Lee E, Wu AH, Hayes RB, Ahn J. 2024. Sociobiome - Individual and neighborhood socioeconomic status influence the gut microbiome in a multi-ethnic population in the US. NPJ Biofilms Microbiomes 10:19. doi:10.1038/s41522-024-00491-y 38467678 PMC10928180

[36] Gacesa R, Kurilshikov A, Vich Vila A, Sinha T, Klaassen MAY, Bolte LA, Andreu-Sánchez S, Chen L, Collij V, Hu S, et al.. 2022. Environmental factors shaping the gut microbiome in a Dutch population. Nature 604:732–739. doi:10.1038/s41586-022-04567-7 35418674

[37] Odamaki T, Kato K, Sugahara H, Hashikura N, Takahashi S, Xiao J-Z, Abe F, Osawa R. 2016. Age-related changes in gut microbiota composition from newborn to centenarian: a cross-sectional study. BMC Microbiol 16:90. doi:10.1186/s12866-016-0708-5 27220822 PMC4879732

[38] Xu C, Zhu H, Qiu P. 2019. Aging progression of human gut microbiota. BMC Microbiol 19:236. doi:10.1186/s12866-019-1616-2 31660868 PMC6819604

[39] Garg K, Mohajeri MH. 2024. Potential effects of the most prescribed drugs on the microbiota-gut-brain-axis: a review. Brain Res Bull 207:110883. doi:10.1016/j.brainresbull.2024.110883 38244807

[40] Simbirtseva KY, O’Toole PW. 2025. Healthy and unhealthy aging and the human microbiome. Annu Rev Med 76:115–127. doi:10.1146/annurev-med-042423-042542 39531852

[41] Gopu V, Camacho FR, Toma R, Torres PJ, Cai Y, Krishnan S, Rajagopal S, Tily H, Vuyisich M, Banavar G. 2024. An accurate aging clock developed from large-scale gut microbiome and human gene expression data. iScience 27:108538. doi:10.1016/j.isci.2023.108538 38230258 PMC10790003

[42] Ma J, Prince A, Aagaard KM. 2014. Use of whole genome shotgun metagenomics: a practical guide for the microbiome-minded physician scientist. Semin Reprod Med 32:5–13. doi:10.1055/s-0033-1361817 24390915

[43] Ranjan R, Rani A, Metwally A, McGee HS, Perkins DL. 2016. Analysis of the microbiome: advantages of whole genome shotgun versus 16S amplicon sequencing. Biochem Biophys Res Commun 469:967–977. doi:10.1016/j.bbrc.2015.12.083 26718401 PMC4830092

[44] Amit G, Bashan A. 2023. Top-down identification of keystone taxa in the microbiome. Nat Commun 14:3951. doi:10.1038/s41467-023-39459-5 37402745 PMC10319726

[45] Weiss AS, Niedermeier LS, von Strempel A, Burrichter AG, Ring D, Meng C, Kleigrewe K, Lincetto C, Hübner J, Stecher B. 2023. Nutritional and host environments determine community ecology and keystone species in a synthetic gut bacterial community. Nat Commun 14:4780. doi:10.1038/s41467-023-40372-0 37553336 PMC10409746

[46] Frioux C, Ansorge R, Özkurt E, Ghassemi Nedjad C, Fritscher J, Quince C, Waszak SM, Hildebrand F. 2023. Enterosignatures define common bacterial guilds in the human gut microbiome. Cell Host Microbe 31:1111–1125. doi:10.1016/j.chom.2023.05.024 37339626

[47] Metwaly A, Reitmeier S, Haller D. 2022. Microbiome risk profiles as biomarkers for inflammatory and metabolic disorders. Nat Rev Gastroenterol Hepatol 19:383–397. doi:10.1038/s41575-022-00581-2 35190727

[48] Phan J, Nair D, Jain S, Montagne T, Flores DV, Nguyen A, Dietsche S, Gombar S, Cotter P. 2021. Alterations in gut microbiome composition and function in irritable bowel syndrome and increased probiotic abundance with daily supplementation. mSystems 6:e0121521. doi:10.1128/mSystems.01215-21 34726487 PMC8562475

[49] Richardson L, Allen B, Baldi G, Beracochea M, Bileschi Maxwell L, Burdett T, Burgin J, Caballero-Pérez J, Cochrane G, Colwell Lucy J, Curtis T, Escobar-Zepeda A, Gurbich Tatiana A, Kale V, Korobeynikov A, Raj S, Rogers Alexander B, Sakharova E, Sanchez S, Wilkinson Darren J, Finn Robert D. 2022. MGnify: the microbiome sequence data analysis resource in 2023. Nucleic Acids Res 51:D753–D759. doi:10.1093/nar/gkac1080 PMC9825492 36477304

[50] Duvallet C, Gibbons SM, Gurry T, Irizarry RA, Alm EJ. 2017. Meta-analysis of gut microbiome studies identifies disease-specific and shared responses. Nat Commun 8:1784. doi:10.1038/s41467-017-01973-8 29209090 PMC5716994

[51] Million M. List of prokaryotes according to their aerotolerant or obligate anaerobic metabolism. Available from: https://www.mediterranee-infection.com/acces-ressources/base-de-donnees/list-of-prokaryotes-according-to-their-aerotolerant-or-obligate-anaerobic-metabolism/. Accessed 17 October 2023

